# Work-Related Musculoskeletal Disorders among Dentists in the United Arab Emirates: A Cross-Sectional Study

**DOI:** 10.3390/medicina58121744

**Published:** 2022-11-28

**Authors:** Amal Hussein, Mahmoud Mando, Ricardas Radisauskas

**Affiliations:** 1 Family and Community Medicine & Behavioral Sciences, College of Medicine, University of Sharjah, Sharjah P.O. Box 27272, United Arab Emirates; 2 Department of Oral Technology, University of Bonn, 53115 Bonn, Germany; 3 Department of Health Management, Faculty of Public Health, Medical Academy, Lithuanian University of Health Sciences, 47181 Kaunas, Lithuania; 4 Department of Environmental and Occupational Medicine, Faculty of Public Health, Lithuanian University of Health Sciences, Tilzes str. 18, 47181 Kaunas, Lithuania; 5 Institute of Cardiology, Lithuanian University of Health Sciences, Sukileliu av. 15, 50162 Kaunas, Lithuania

**Keywords:** work-related musculoskeletal disorders, dentists, private practice, governmental practice, ergonomics

## Abstract

*Background and Objectives:* A dental environment possesses a serious ergonomic health issue on the dental team members which in turn contributes to the development of work-related musculoskeletal disorders (WMSDs). The present research is aimed at evaluating the frequency of musculoskeletal disorders and their associated sociodemographic and work environment risk factors among dentists in the United Arab Emirates. *Material and Methods:* In this cross-sectional study, a pretested and validated questionnaire was sent via email as well as on different social media platforms to a total of 497 dentists. A total of 179 dentists completed the study survey, with a total response rate of 36%. A binary logistic regression model was conducted to identify significant risk factors associated with WMSDs. *Results:* Dentists in the United Arab Emirates experience a high prevalence of work-related musculoskeletal disorders (90.4%) which were associated with high levels of stress in the work environment. Furthermore, dentists in the private sector worked more clinical hours per day (*p* = 0.000) and had more financial stress (*p* = 0.007) as compared to those in the governmental sector. Gender (*p* = 0.007), age (*p* = 0.01), monthly income (*p* = 0.073), work experience (*p* = 0.037), number of patients treated per day (*p* = 0.049), and the use of an ergonomic dental chair (*p* = 0.005) were all factors associated with a greater number of affected body regions by WMSDs. Multivariate binary logistic regression for the number of regions affected by WMSDs revealed that not using an ergonomic dental chair (OR 2.70, 95% CI, 1.14–6.36) and high stress in the work environment (OR 1.31, 95% CI 1.02 to1.67) were associated with more body regions being affected by WMSDs. *Conclusions:* This study highlights the high prevalence rate of WMSDs among dentists in the UAE. Future research should be directed towards reducing stress in the work environment, increasing awareness regarding the importance of an ergonomic dental chair, and reducing gaps between private and governmental practices.

## 1. Introduction

### Background

People working in the dental field, including dentists, are subjected to numerous occupational health hazards including biological, physical, psychological, and chemical [1]. In addition, the dental environment possesses a serious ergonomic health impact on the dental team members, leading to the development of work-related musculoskeletal disorders (WMSDs) [2]. Musculoskeletal disorders are a group of disorders which affect the musculoskeletal system including nerves, muscles, blood vessels, ligaments, tendons, bones, joints, and vertebral discs [3]. The prevalence of musculoskeletal disorders among dentists is 95.8% in Germany [4], 64% in Australia [5], 54% in Sweden [6], 60% in Poland [7], and 60% in Denmark [8]. Furthermore, according to a systematic review conducted in 2009, the worldwide prevalence of musculoskeletal disorders among dental professionals ranges between 64 and 93% [9]. The occurrence of musculoskeletal disorders among dental professionals is well-documented in the literature and the causes of such disorders can be attributed to several factors including abnormal posture, repetitive movement of hands, forceful movement of hands, standing or sitting for a long time, stress, absence of magnification tools and proper lighting, and genetics [3,9,10]. Symptoms of musculoskeletal disorders appear early in dental professionals due to the high number of clinical hours during clinical training [11]. The symptoms of musculoskeletal disorders include tingling, stiffness, burning, numbness, discomfort, and fatigue. On the other hand, the signs include a decrease in grip strength, a decrease in range of motion, and a loss of normal sensation and coordination [12]. Pain associated with MSDs in dental professionals may occur in various body regions including the upper back, lower back, neck, fingers, wrist, shoulders, feet, knees, and hips. In one study, the majority of dental professionals reported back pain as their main experienced symptom [9]. 

Several studies have been conducted to assess the prevalence of work-related musculoskeletal disorders and their associated risk factors in various countries [10,11]. In the United Arab Emirates (UAE), few studies have investigated the prevalence of MSDs and the general health status of dental professionals. A study reported a high prevalence of pain due to MSDs, yet variations in the prevalence as well as the regions of MSD-induced pain occur among different dental specialists. In general, dentists in the UAE were found to have adopted an unhealthy lifestyle, as more than 60% of them reported not exercising regularly [13] and around 15% were smokers [14]. However, these studies were conducted more than five years ago and, to our knowledge, no recent research has investigated the prevalence of MSDs in dentists during the COVID-19 pandemic. Therefore, the present research aimed at evaluating the frequency of musculoskeletal disorders and their associated sociodemographic and work-related environmental risk factors among dentists in the United Arab Emirates, during the COVID-19 pandemic period.

## 2. Materials and Methods

### 2.1. Ethics

This study was reviewed and approved on 27 May 2021 by the Research Ethics Committee at the University of Sharjah before commencing field work (reference number: REC-21-05-26-02-S).

In this cross-sectional study, dentists practicing in the UAE were recruited from governmental and private dental clinics.

### 2.2. Sample, Inclusion and Exclusion Criteria

The sample was selected based on a non-probability convenience sampling method. Email addresses of participants were selected from the list of registered dentists at the Ministry of Health’s website in the UAE. Additionally, the heads of departments in dental hospitals were contacted personally and provided with a link to the survey and then were asked to distribute the link to their colleagues. Furthermore, dentists were approached on LinkedIn and were sent the link to the survey as a chat message. The minimum sample size needed to conduct this study was calculated using the Cochrane formula for calculating sample size in cross-sectional studies (*n* = 1.96^2^ × *p* × (1 − *p*)/error^2^) [15], where (*n*) is the minimum sample size needed for a 95% confidence interval and there is an error of 5% and expected prevalence (P) of MSDs in dental professionals of 70% [16]; the minimum sample size needed to conduct this study was 323 dentists.

Inclusion and exclusion criteria were applied to identify eligible study participants. Practicing dentists who had more than three years of experience in the UAE were eligible to participate in the study. Dentists who suffered from any systemic disease that might cause musculoskeletal pain, and those with 3 years of experience or less, were excluded from this study.

### 2.3. Data Collection Instruments

A pretested and validated online questionnaire [17] was sent to all dentists via email and was also distributed on different social media platforms. Survey reminders were sent to dentists to increase their response rate. The questionnaire included a total of 31 questions that were organized into four different sections. The first section was a general dentist survey that included questions about sociodemographic variables such as age, gender, marital status, and monthly income in addition to questions concerning height, weight, and specialty of participants. The second section was specifically directed towards the work environment of clinical practice including type of practice, years of experience, environmental stress, number of patients seen per day, number of working hours, scheduled breaks, the use of an ergonomic chair, dental loupes, and appropriate lighting. Participants were also asked to report, on a scale from 0 to 10, the stress level that they experience on a daily basis due to their work environment, financial situation, and personal life issues. The third section included questions regarding the presence of pain, discomfort, numbness, type, location, and intensity of pain as well as actions taken to prevent or alleviate the musculoskeletal symptoms. The visual analogue scale (VAS), from 1 to 10, was used to measure the intensity of pain. Furthermore, the prevalence of musculoskeletal disorders among dentists was measured in the fourth section of the questionnaire using a modified version of the Standardized Nordic musculoskeletal questionnaire [18], previously described by Kuorinka et al. [19]. This questionnaire has been previously validated and frequently used in several studies [18,20]. The body areas presented in the questionnaire were ankle/feet, knees, hips/thighs, lower back wrists/ hands, elbows, upper back, shoulders, and neck [18,19].

Dentists practicing in the UAE sit for a licensing exam which is administered in the English language. Thus, the study questionnaire and the participant information sheet (PIS) were prepared only in the English language. The questionnaire was anonymous, as participants were not asked to record any identifying information. Therefore, withdrawal from the study was possible only while filling out the questionnaire.

This study has used implied consent as signatures were not required by the study participants. Before accessing the questionnaire link, participants read the PIS that included all the necessary details about the study. Opening the link and filling out the questionnaire indicated a participant’s consent to participate in the study. Only the investigators had access to the collected data.

### 2.4. Statistical Analysis

Collected data were first imported to Microsoft Excel 2016 and then transferred to the Statistical Package for Social Sciences (SPSS) software version 27.0. Armonk, NY (IBM Corp, 2020) [21] for data analysis. Descriptive statistics including medians, means, percentages, and frequencies were used to summarize and condense data. The Kolmogorov–Smirnov test was used to check for the normality of the scale data. Median values were used to categorize scale variables that showed skewed distribution. Bivariate analysis using the Chi-square (χ^2^) test was conducted to explore associations between categorical variables. For example, we studied the association between the number of body regions affected by musculoskeletal disorders (0 body regions, 1–2 body regions, and >2 body regions) and the different work environments as well as sociodemographic correlates. A Mann–Whitney test was used to compare quantitative work environment risk factors between the different types of dental practices. Binary logistic regression model, using the enter method, was performed to identify significant predictors of the dependent variable, which was the number of body regions affected by WMSDs. To conduct the regression model, the dependent variable was categorized into two groups, ≤2 body regions and >2 body regions affected by WMSDs among dentists. Factors that were statistically significant in the bivariate analysis (*p*-value < 0.05) and those showing a significance level below 0.20 were included in the multivariate binary logistic regression model. Prior to performing the logistic regression model, the assumptions of multicollinearity and multivariate outliers were checked using the variance inflation factor (VIF < 10) and Mahalanobis distance, respectively. The Homser and Lemeshow test and the Link test were used to verify the adequacy of the model and the specificity of the regression model, respectively. The estimates of the strengths of associations were demonstrated by the odds ratio (OR) with its 95% confidence interval (CI). A two-tailed *p* < 0.05 was considered statistically significant.

## 3. Results

A total of 179 dentists completed the study survey, with a total response rate of 36%; however, 22 were excluded from the study sample due to their short work experience (less than three years). Consequently, a total of 157 dentists comprised the final study sample, with 55.4% (*n* = 87) working in the private sector and 38.9% (*n* = 61) from the governmental sector. The main sociodemographic characteristics of the study population are summarized in Table 1. Of all study participants, 49.7% (*n* = 78) were general dentists while 50.2% (*n* = 79) were specialists, 45.2% (*n* = 71) were males, 47.8% (*n* = 74) were aged between 25 and 34 years, 79% (*n* = 124) were married at some point, and 52.2% (*n* = 82) had an income above AED 20,000.

The median work experience among dentists was 9 years, while 43.9% of dentists had experience of 9 years or less and 56.1% had more than 9 years of experience.

The prevalence of work-related musculoskeletal disorders among dentists was 90.4%. The most affected body region in terms of pain, as reported by study participants, was the neck (58.8 %), followed by the lower back (55.4%), shoulders (50.7%), upper back (28.4%), wrists and hands (26.4%), hips and thighs (10.8%), knees (8.1%), and elbows (6.8%) (Table 2). Furthermore, 42.7% of participants had more than two while 57.3% had one or two affected body regions. Of participants with musculoskeletal disorders, 29.3% suffered from acute pain, 46% reported chronic pain, and 21.7% stated that they had no pain. The severity of pain, as measured by VAS, had a median score of 5 (IQR: 7–8).

There was a statistically significant difference between the number of body regions affected by WMSDs and gender (*p* = 0.007): 52.3% of female dentists compared to 31% of male dentists reported more than two affected body regions, as seen in Table 3. Similarly, the number of body regions affected by WMSDs were significantly different between age groups (*p* = 0.01). Among dentists who were less than 35 years old, 53.3% reported more than two affected body regions as compared to 32.9% among those who were 35 years old or more. On the other hand, BMI (*p* = 0.782), marital status (*p* = 0.26), and monthly income (*p* = 0.073) were not statistically associated with the number of affected body regions. Similarly, being a general or specialist dentist did not show a statistically significant association (*p* = 0.127) with the number of affected body regions. The percentage of general dentists with more than two affected body regions was 50% compared to 35.4% of specialists with more than two affected body regions.

The use of an ergonomic dental chair showed a statistically significant association with the number of affected body regions (*p* = 0.005). Having work experience of 9 years or fewer, or more than 9 years, was significantly associated with the number of affected body regions (*p* = 0.037): 53.6% of dentists with nine years or fewer of experience had more than two affected body regions as compared to 34.1% among dentists with more than nine years of experience. Similarly, there was a statistically significant association between the number of patients seen per day by dentists and the number of affected body regions (*p* = 0.049). Of dentists who see more than eight patients per day, 51.2% reported pain in more than two body regions as compared to 33.3% in those who see fewer than eight patients daily.

A percentage of 37.6% of participants took medications to relieve the symptoms of WMSDs, while 55.9% of dentists who suffer from more than two affected body regions reported the consumption of medications compared to 42.4% in those who suffer from fewer than two affected body regions. Furthermore, 25.5% of participants took a sick leave because of WMSDs. An amount of 55% of dentists who suffer from more than two affected body regions took sick leaves and 45% of those with fewer than two affected body regions took sick leaves. Furthermore, 60.4% of dentists in private practices who suffered from two or fewer affected regions reported that they exercise as compared to 56.3% with more than two regions (*p* = 0.028), as seen in Table 4. On the other hand, there was no statistically significant difference between physical activities and the number of affected regions among dentists at governmental practices (*p* = 0.305), as seen in Table 5.

There was no statistically significant difference between the number of patients seen per day (*p* = *0*.096), scheduled breaks (*p* = 0.68), quality of dental unit lighting (*p* = 0.99), use of magnification loupes (*p* = 0.66), seating position while working (*p* = 0.88), the use of an ergonomic dental chair (*p* = 0.99), and the type of dental practice.

A statistically significant association was found between clinical training and education about ergonomics and the type of practice (*p* = 0.04): 68.9% of dentists who did not receive clinical training and education about ergonomics were working in the private sector and the remaining 31.1% were from the governmental sector.

The results of the Mann–Whitney U test as depicted in Table 6 showed a statistically significant relationship between stress from one’s financial situation and the type of practice (*p* = 0.01). This can be attributed to the statistical significant difference in the monthly income between dentists in private and governmental practices (*p* < 0.001). On the other hand, there was no statistically significant difference between stress from one’s work environment (*p* = 0.082) or stress from personal life (*p* = 0.937) and the type of dental practice. Additionally, there was a statistically significant difference in the number of clinical hours per day and the type of practice (*p* =0.00). The majority of dentists in private practices (80.5%) worked up to 8 h per day compared to 55.6% of dentists in the governmental sector who worked up to 8 h per day.

In order to identify the significant predictors of having more than two body regions affected by WMSDs, while controlling for potential confounders, a binary logistic regression model was used. The dependent variable was the number of regions affected by WMSDs (0 = ≤2 body regions and 1 = >2 body regions) while the independent variables were the dentist’s age, gender, specialty, type of practice, experience, clinical hours per day, number of patients per day, the use of dental magnification loupes, an ergonomic dental chair, stress in the work environment, and stress from personal life. The results of the multivariate binary logistic regression for predicting the number of regions affected by WMSDs are presented in Table 7. Results have shown that not using an ergonomic dental chair (OR 2.70, 95% CI, 1.14–6.36) and high stress in the work environment (OR 1.31, 95% CI 1.02 to 1.67) were the only two significant predictors of reporting more than two body regions affected with pain due to MSDs among dentists. Based on our model, the explained variation in the outcome variable ranges between 20.6% and 27.7% according to the Cox and Snell R^2^ or Nagelkerke R^2^, respectively.

## 4. Discussion

This study has investigated the sociodemographic and work environment risk factors of musculoskeletal disorders among dentists in the United Arab Emirates. Additionally, this is the first study to compare the difference in prevalence and characteristics of musculoskeletal disorders between practicing dentists in the private sector and those in the governmental sector.

Among all sociodemographic correlates, gender and age were the only risk factors which were significantly associated with the number of body regions affected by WMSDs. The relationship between gender and WMSDs are in line with other studies that reported a gender difference in relation to the presence of musculoskeletal disorders [3,8]. Additionally, dentists aged less than 35 years old had more than two affected body regions when compared to those who were 35 years old or more. On the other hand, several studies reported that the prevalence of WMSDs was positively associated with older age [22,23]. Our results can be explained by the fact that the older the dentist, the more he is aware of the consequences of WMSDs and methods of prevention. Similarly, the results of this study revealed that dentists with more than 9 years of experience had a smaller number of regions affected by WMSDs.

Among the work environment risk factors, the use of an ergonomic dental chair, the number of years of experience, number of patients seen per day by dentist, and medication intake to relieve work-related WMSDs showed statistically significant associations with the number of affected body regions. Similarly, a study reported that students who used an ergonomic dental chair adopted a more favorable posture than students who used a conventional dental chair [24]. Additionally, a study conducted in China reported a significant association between the time spent treating each patient and the occurrence of MSDs in the neck, shoulders, and hand-rest regions [25]. Moreover, stress in the work environment was positively related to the increase in the number of affected body regions. These results are consistent with those reported by previous studies, which have emphasized the significant associations between the level of stress and work-related musculoskeletal disorders [24,26,27,28].

There was a statistically significant association between clinical training and education about ergonomics and the type of practice. Additionally, 40.8% of dentists did not receive clinical training and education about ergonomics while studying at university. These results are in line with a study that was conducted in Australia. This study reported that only 30% of general dentists have received education about ergonomics in universities [17]. These results emphasize the lack of structured education about ergonomics during undergraduate studies. Furthermore, the results of this study have revealed that dentists who work in the private sector have more financial stress as compared to those who work in the governmental sector. The increase in the financial stress among dentists in the private sector can be attributed to their lower monthly income as compared to dentists in the governmental sector.

Additionally, there was a statistically significant association between the type of practice and number of clinical hours per day. In other words, dentists in private practices work more clinical hours per day and have a lower monthly income as compared to those in governmental practices. Similarly, a study that was conducted in Brazil revealed that 70% of dentists who work in private clinics work approximately 12 h daily, thus having a greater working load compared to dentists who work in public clinics [29].

A study published by Al-Rawi et al. investigated only the prevalence of musculoskeletal pain among dentists in the United Arab Emirates. However, the result of this study is to be questioned since musculoskeletal disorders include symptoms other than pain such as tingling, stiffness, burning, numbness, discomfort, and fatigue. Additionally, this study did not investigate the risk factors of WMSDs among dentists or the difference between governmental or private sectors. Furthermore, this study used the Standardized Nordic musculoskeletal questionnaire, which does not only address musculoskeletal pain. Instead, it includes other symptoms which were not reported in the Al-Rawi study [13]. Similarly, Hashim et al. investigated the prevalence of musculoskeletal symptoms among dental students in the UAE. However, the author reported only musculoskeletal pain without considering other symptoms. This study has highlighted a high prevalence of 68.3% of WMSDs among students [30], which can be attributed to the lack of education and clinical training about ergonomics during dental school. This can also be reflected in the results of our study, since 40.8% of participants stated that they have not received such training.

Hashim et al. has investigated health problems among dentists in the UAE [14]. Despite the well documented high prevalence of WMSDs among dentists, this study has not mentioned anything about the prevalence of WMSDs among dentists in the UAE. Therefore, different aspects of the health of dentists in the UAE have still not been fully investigated. This study has also shown that more than half of the dentists in the UAE do not exercise regularly. The results of this study are in line with ours, since more than 60% of dentists in our study do not exercise. In line with these results, several studies have reported that a high prevalence of WMSDs among dentists was associated with a lack of physical activities. A study that was conducted in China reported that over 60% of Chinese dentists practice regular physical exercises, with an average frequency of 2.2 times per week, and that regular physical exercise was associated with less neck pain [25]; thus, practicing regular physical activities helps to reduce the symptoms of WMSDs and improve the quality of life.

The results of this study are in line with other studies, which reported a high prevalence of work-related musculoskeletal disorders among dentists, as high as 88.9% in Australia [15] and 92% in Sweden [6]. Furthermore, the three most affected body regions by WMSDs in the present study were the neck, lower back, followed by the shoulders. These results were similar to other studies that reported these three body regions as the most affected regions by WMSDs [8,19]. On the other hand, some studies reported a prevalence of 67% who experienced lower back pain, followed by the neck (40–67%) and shoulders (15–20%) [8,31,32]. In other words, the three most affected regions by WMSDs in the literature are the neck, lower back, and shoulders. This may be attributed to the high work strain exerted on these three body regions while working intra-orally, including an improper seating position with a tilted pelvis, forward tilting of the neck, and unrelaxed repetitive shoulder movement.

In the present study, there was no statistically significant relationship between the use of dental magnification loupes and the number of regions affected by WMSDs. However, two studies that investigated the impact of working with dental loupes on the level of musculoskeletal discomfort among dental hygienists have reported a significant reduction in the level of discomfort in the arm, shoulder, and hand regions but no statistical significance pertaining to pain reduction in the neck region [33,34].

This study has a few limitations that are mainly related to its research methodology. The actual number of study participants, 179, was much lower than the calculated minimum needed sample size (323). The small sample size is a potential threat to the external validity of the study results. Another limitation in this study is related to its low response rate (36%) which highlights the drawbacks of responder bias when collecting data using an online survey. Therefore, future studies are recommended to collect their data using face-to-face personal interviews with dentists in order to increase response rate and eventually reach the minimum needed sample size. Moreover, this study is prone to the healthy worker effect bias. An underestimation of the actual prevalence of WMSDs is not unexpected as dentists who suffer from WMSDs may fail to report this due to their absenteeism during the data collection period. Another limitation may arise from the cross-sectional nature of the study’s research design where causal relationships could not be identified. Furthermore, this study was conducted during the COVID-19 pandemic. Due to the pandemic, more efforts were carried out to recruit participants on social media or in person when possible. However, contact was only possible with heads of departments. Unfortunately, most of our responses were collected through the mailed questionnaire which subjects the following study to selection bias or nonresponse bias. Moreover, this study did not report the severity of symptoms but only the number of affected body regions by musculoskeletal disorders. Future studies should investigate the psychosocial and organizational factors which may contribute to the development of musculoskeletal disorders as suggested by Bodin et al. [35].

Due to the low number of participants in different dental specialties, we had to combine these specialties under one category, which is specialist dentists. Therefore, future studies should aim to identify the prevalence of WMSDs in different dental specialties. In this way, specialist dentists can understand the negative consequences associated with each specialty and those who work in high-risk specialties can minimize the stress in their work environment and use an ergonomic dental chair to avoid future development of WMSDs. In the end, it would be crucial to note that the use of logistic regression is limited by itself, as it does not allow authors to analyze the direct and indirect influence of risk factors on WRMDs [36,37]. Thus, future studies should choose to analyze the relationship between risk factors and WMSDs through structural equation modeling.

## 5. Conclusions

Dentists in the United Arab Emirates experience a high prevalence rate of work-related musculoskeletal disorders, which are associated with high levels of stress in the work environment. The increased levels of stress are positively related to the increased number of affected body regions with WMSDs.

The results of the binary logistic regression model for predicting the number of regions affected by WMSDs revealed that not using an ergonomic dental chair and high stress in the work environment were associated with more body regions being affected by WMSDs. On the other hand, there was no statistically significant difference between exercising and the number of affected body regions.

There was no statistically significant difference between stress from the work environment or stress from personal life and the type of dental practice. Additionally, there was a statistically significant difference in the number of clinical hours per day and the type of practice. The majority of dentists in private practices (80.5%) worked up to 8 h per day compared to 55.6% of dentists in the governmental sector who worked up to 8 h per day. Furthermore, dentists in the private sector had a lower monthly income and more financial stress as compared to those in the governmental sector. Therefore, future studies should aim to reduce gaps between private and governmental practices. Moreover, there was no statistically significant difference between exercising and the type of dental practice. However, within the private sector, there was a statistically significant association between the number of regions with WMSDs and physical activities.

## Figures and Tables

**Table 1:** Sociodemographic and other characteristics of the study population (*n* = 157).

Variables		*n* (%)
Gender	Males	71 (45.2)
	Females	86 (54.8)
Age groups	<35	74 (47.8)
	≥35	83 (52.2)
Marital status	Ever married	124 (79)
	Never married	33 (21)
BMI	<18.5	2 (1.3)
	18.5 to 24.9	72 (45.9)
	25 to 29.9	55 (35)
	30+	28 (17.8)
Income	Less than AED 20,000	75 (47.8)
	More than AED 20,000	82 (52.2)
Specialty	General dentists	78 (49.7)
	Specialist	79 (50.3)
Years of experience	≤9 years	69 (43.9)
	>9 years	88 (56.1)
Number of patients/day	≤8 patients	75 (47.8)
	>8 patients	82 (52.2)
Clinical hours/day	8 h	84 (54.1)
Type of practice	Private	87 (55.4)
	Governmental	61 (38.9)
	Both	9 (5.7)

BMI (Body Mass Index), AED (Emirati Dirham).

**Table 2:** The frequency of affected body regions by MSDs.

Body Region	Total *n* (%)	Private *n* (%)	Governmental *n* (%)	*p* Value
Neck	87 (58.8)	5 (57.5)	37 (60.7)	0.698
Lower back	82 (55.4)	48 (55.2)	34 (55.7)	0.946
Shoulders	75 (50.7)	36 (41.4)	39 (63.9)	0.007
Upper back	42 (28.4)	25 (28.7)	17(27.9)	0.908
Wrists and hands	39 (26.4)	23 (26.4)	16 (26.2)	0.978
Hips and thighs	16 (10.8)	7 (8)	9 (14.8)	0.196
Knees	12 (8.1)	8 (9.2)	4 (6.6)	0.563
Elbows	10 (6.8)	6 (6.9)	4 (6.6)	0.936

**Table 3:** Frequency of WMSD by sociodemographic and work-related characteristics (*n* = 157).

Variables		No	≤2 WMSD	>2 WMSD	Significance Chi-Square, df, *p*-Value
		*n* (%)	*n* (%)	*n* (%)	
Gender	Males	11 (15.5)	38 (53.5)	22 (31)	9.8, 2, 0.007
	Females	4 (4.7)	37 (43)	45 (52.3)	
Age groups (years)	<35	3 (4)	32 (42.7)	40 (53.3)	9.2, 2, 0.01
	≥35	12 (14.6)	43 (52.4)	27 (32.9)	
Marital status	Never married	1 (3)	15 (45.5)	17 (51.5)	2.6, 2, 0.26
	Ever married	14 (11.3)	60 (48.4)	50 (40.3)	
BMI (kg/m^2^)	<18.5	0 (0)	0 (0)	2 (100)	3.2, 6, 0.782
	18.5–24.9	6 (8.3)	36 (50)	30 (41.7)	
	25–29.9	6 (10.9)	25 (45.5)	24 (43.6)	
	30>	3 (10.7)	14 (50)	11 (39.3)	
Income (AED)	<AED 20,000	3 (4)	39 (52)	33 (44)	5.2, 2, 0.073
	≥AED 20,000	12 (14.6)	36 (43.9)	34 (41.5)	
Specialty	General dentists	5 (6.4)	34 (43.6)	39 (50)	4.1, 2, 0.127
	Specialist	104 (12.7)	12 (51.9)	28 (35.4)	
Years of experience	≤9 years	4 (5.8)	28 (40.6)	37 (53.6)	6.6, 2, 0.037
	>9 years	11 (12.5)	47 (53.4)	30 (34.1)	
Number of patients/day	≤8 patients	10 (13.3)	40 (53.3)	25 (33.3)	6, 2, 0.049
	>8 patients	5 (6.1)	35 (42.7)	42 (51.2)	
Type of practice	Private	7 (8)	48 (55.2)	32 (36.8)	4.3, 2, 0.112
	Governmental	7(11.5)	23 (37.7)	31 (50.8)	
Dental magnification loupes	No	10 (9.5)	55 (52.4)	40 (38.1)	2.9, 2, 0.227
	Yes	5 (9.6)	20 (38.5)	27 (51.9)	
Ergonomic dental chair	No	5 (4.8)	48 (46.2)	51 (49)	10.5, 2, 0.005
	Yes	10 (18.9)	27 (50.9)	16 (30.2)	
Dental lighting	No	11 (16.2)	31 (45.6)	26 (38.2)	6.1, 2, 0.046
	Yes	4 (4.5)	44 (49.4)	41 (46.1)	
Awareness of seating position	No	4 (6.3)	33 (51.6)	27 (42.2)	1.5, 2, 0.457
	Yes	11 (11.8)	42 (45.2)	40 (43)	
Sick leaves	No	15 (12.8)	57 (48.7)	45 (38.5)	7.1, 2, 0.028
	Yes	0 (0)	18 (45)	22 (55)	
Consumption of medications	No	14 (14.3)	50 (51)	34 (34.7)	10.5, 2, 0.005
	Yes	1 (1.7)	25 (42.4)	33 (55.9)	
Exercising	No	2 (3.4)	30 (50.8)	27 (45.8)	4.1, 2, 0.125
	Yes	13 (13.3)	45 (45.9)	40 (40.8)	

**Table 4:** A comparison of physical activities and the number of regions affected with WMSDs among dentists in private practices.

Exercising	Private Practice *n* (%)			Significance Chi-Square, df, *p*-Value
	0 Regions with WMSDs	1–2 Regions with WMSDs	>2 Regions with WMSDs	
Yes	7 (100%)	29 (60.4%)	18 (56.3%)	7.2, 2, 0.028
No	0 (0.0%)	19 (39.6%)	14 (43.8%)	

**Table 5:** A comparison between physical activities and the number of regions affected with WMSDs among dentists in governmental practices.

Exercising	Governmental Practice *n* (%)			Significance Chi-Square, df, *p*-Value
	0 Regions with WMSDs	1–22 Regions with WMSDs	>2 Regions with WMSDs	
Yes	6 (85%)	13(56.5%)	18 (58.1%)	**2.1, 2, 0.305**
No	1 (14.3%)	10 (43.5%)	13 (41.9%)	

**Table 6:** A comparison of work environment risk factors between private and governmental practice.

Risk Factor	Private Sector Median Value	Governmental Sector Median Value	U *	Z	*p*-Value
Clinical hours	8	7	1527	−4.856	0.000
Stress from work environment	7	8	2212	22,121.741	0.082
Stress from financial situation	7	5	1969.5	−2.682	0.007
Stress from personal life	6	6	2633.5	–0.078	0.937

* The non-parametric Mann–Whitney U test was used to compare risk factors between private and governmental sectors as the risk factors showed a non-normal distribution of data.

**Table 7:** A binary logistic regression model was used to identify predictors of >2 body regions affected by WMSDs by sociodemographic and work environment risk factors.

Variable		b	SE(b)	OR (95% CI)	*p*-Value
**Dependent Variable = Number of Regions Affected by WMSDs1 (*n* = 157)** **(0= ≤ 2 body regions and 1 = >2 body regions)**					
Age	<35 years old ≥35 years old	0.048	0.589	1.04 (0.33–3.32)	0.936
Gender	Male Female	- 0.687	- 0.405	1 1.98 (0.89–4.39)	- 0.090
Specialty	General dentist Specialist	0.139 -	0.469 -	1.15 (0.458–2.884) 1	0.767 -
Type of practice	Private Governmental	- 0.469	- 0.410	1 1.59 (0.715–3.569)	- 0.253
Experience	≤9 >9	- 0.951	- 0.591	1 2.58 (0.81–8.24)	- 0.108
Clinical hours/day		-0.003	0.073	0.99 (0.86–1.15)	0.967
Number of patients/day	≤8 Patients >8 Patients	- 0.467	- 0.406	1 1.59 (0.72–3.53)	- 0.249
Dental magnification loupes	No Yes	- 0.519	- 0.424	1 1.68 (0.73–3.86)	- 0.221
Ergonomic dental chair	No Yes	0.994 -	0.437 -	2.70 (1.14–6.36) 1	**0.023** -
Stress in work environment		0.273	0.125	1.31 (1.02–1.67)	**0.029**
Stress from personal life		0.038	0.091	1.04 (0.86–1.24)	0.678

Model fit: Hosmer and Lemeshow test χ^2^ (8, *n* = 157) = 11.35, *p* = 0.18–2 log likelihood 164.697. ^1^ Work-related musculoskeletal disorders.
